# Development of screening questions for doctor–patient consultation assessing the quality of life and psychosocial burden of glioma patients: an explorative study

**DOI:** 10.1007/s11136-021-02756-x

**Published:** 2021-01-31

**Authors:** Hannah Voß, Peter Scholz-Kreisel, Christoph Richter, Florian Ringel, Susanne Singer, Mirjam Renovanz

**Affiliations:** 1 Department of Neurosurgery, University Medical Center, Johannes-Gutenberg-University Mainz, Mainz, Germany; 2 Division of Epidemiology and Health Services Research, Institute of Medical Biostatistics, Epidemiology and Informatics, University Medical Center, Johannes-Gutenberg-University Mainz, Mainz, Germany; 3 Department of Neurooncology, University Hospital, Tuebingen, Hoppe-Seyler-Str. 3, 72076 Tuebingen, Germany; 4 Department of Neurosurgery, University Hospital, Tuebingen, Hoppe-Seyler-Str. 3, 72076 Tuebingen, Germany

**Keywords:** Brain tumor, Quality of life, Distress, Glioma, Neurooncology, Screening

## Abstract

**Purpose:**

Psychosocial screening for glioma patients is challenging because many patients suffer from neurocognitive deficits, which may impair assessment. This study’s aim was to exploratively develop three screening questions for unmet needs to prospectively be applicable in patient–doctor consultation.

**Methods:**

Patient interviews, a survey for health-care professionals and a weighted scoring procedure were developed for this study. Six main areas were defined according to main areas of validated questionnaires (psyche, cognition, body, role functioning, social support, unmet needs). Patients and health-care professionals rated the importance of these areas and corresponding items, patients additionally stated whether the issues addressed affected them.

**Results:**

A total of 50 patients were included, and 36 health-care professionals participated in the online survey. The three areas (psyche, body and cognition) considered to be most relevant by both, health-care professionals and patients, generated three screening questions. If the patient was affected by the issue addressed with a screening question, a subordinate question from that area that our patient sample considered most important could additionally be asked. The elaborated screening questions are the following: (1) main area psyche: “Has your mood worsened?”, (2) main area body: “Do physical changes put a strain on you?”, and (3) main area cognition: “Has your memory capacity worsened?”

**Conclusion:**

These questions represent a basis for further research regarding their application in neuro-oncological clinical routine.

**Supplementary Information:**

The online version of this article (10.1007/s11136-021-02756-x) contains supplementary material, which is available to authorized users.

## Background

Gliomas compose 26% of all primary brain tumors and 81% of malignant CNS tumors with unfavorable prognoses [1]. Patients with gliomas should be treated with a multidisciplinary approach whereby their quality of life needs to be considered [2–4]. Focusing on glioma patients’ often diminished quality of life in clinical routine can strengthen i. a. doctor–patient communication, participation and satisfaction with treatment [5, 6].

Since these patients also suffer from relevant distress, frequent screening is indispensable [7, 8]. The term “distress” hereby describes a negative emotional state arriving from various biopsychosocial factors [9] that can influence not only a patients’ quality of life [10] but also their adherence to treatment [11].

Glioma patients in unfavorable clinical conditions are at risk of having unmet needs [12]. Against the background that not all patients who are distressed wish to receive support and vice versa, assessing unmet needs additionally to distress screening could give information on their need of support [13].

In self-assessments, however, a high nonresponse rate could be specific for glioma [14] since various physical and neuropsychological symptoms can complicate the use of screening instruments [15]. It is possible that the true rates of distress, mental and cognitive problems are higher than reported [16]. The development of a practical screening procedure for glioma should be considered [17]. As many patients, especially those in the late disease trajectory, are not able to complete any questionnaires or handle tablets, questions directly asked during a patient–doctor consultation might be helpful: Especially in this situation, it is not enough to leave providing support to health-care professionals’ intuition. Therefore, predefined screening questions are needed in order to systematically detect unmet needs. The aim of this study therefore was to develop three screening or signaling questions adapted for glioma patients potentially be asked either during a doctor–patient consultation or by health-care professionals in general.

## Methods

### Study design

The explorative study’s aim was to find out which screening questions are considered important by both glioma patients and health-care professionals to be prospectively tested in further research. First, considering literature and three highly established screening questionnaires, we developed the interview described below. The literature we are referring to is provided in detail in the section “interview construction”.

After a pretest in 10 patients, the standard operating procedure for the interview was optimized. Thereafter, patients in the neuro-oncological outpatient department fulfilling the following inclusion criteria were invited to participate: (1) intracranial located glioma [1], (2) understanding of the German language, (3) ability to give informed consent, and (4) absence of aphasia.

The exclusion criteria were (1) diagnosis other than glioma, (2) non-cranial localization of the tumor, (3) lack of informed consent, and (4) inability to answer interview questions. Sociodemographic and clinical data were assessed with a questionnaire.

**Fig. 1:**
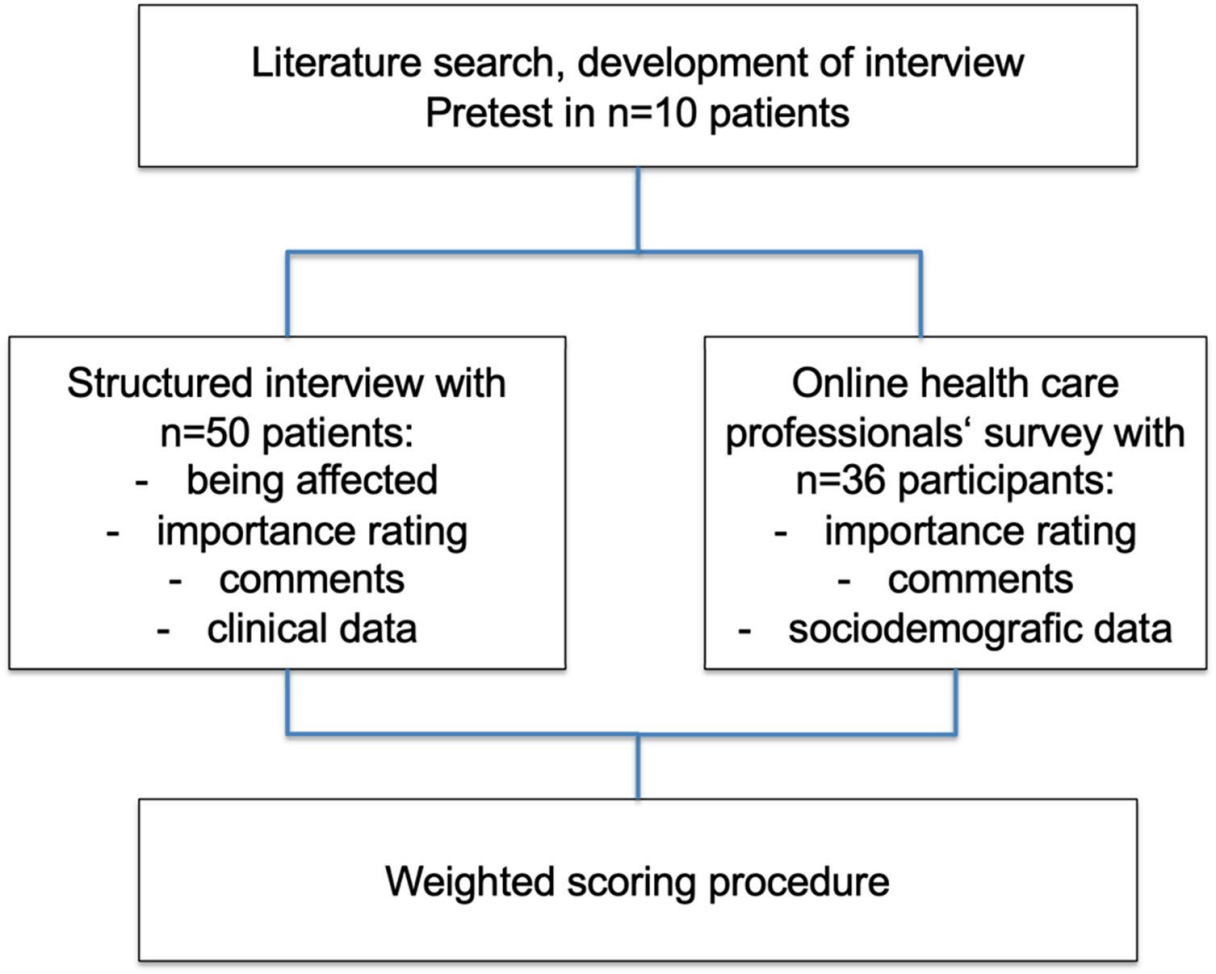
Study design and workflow

Simultaneously, an online survey with health-care professionals was conducted. All members (*n* = 350) of the Neurooncology Working Group of the German Cancer Society were contacted via mail and supplied with a link to the anonymous survey. Being a doctor was the inclusion criterion (the participants provided information on their professions in the survey). Figure 1 provides an overview over the course of the study.

### Interview construction

The interview developed for this study consists of six areas with several items each that are considered relevant for glioma patients according to literature (see below). Quality of life assessment and psychosocial screening with instruments may be sometimes too complex and signaling items of questionnaires are helpful for clinical practice [21]. We therefore focused on instruments which have been applied in glioma patients or have been validated in brain tumor patients: the European Organization for Research and Treatment of Cancer Core Quality of Life Questionnaire (EORTC QLQ-C30) and brain module (BN20) [18, 19], which was developed for brain tumor patients assessing health-related quality of life (HRQoL), and the Distress Thermometer (DT) along with the associated problem item list [11]. The interview’s area “unmet needs” asks for the need for support from professions and sources that are included in the Patient’s Perspective Questionnaire (PPQ) which is focusing on unmet needs [12]. The questionnaires themselves were not applied in this study. We collected the items of the questionnaires and defined exploratively main areas, which represent domains probably relevant for glioma patients.

The derived interview areas according to the questionnaires and instruments are: (1) *Psyche* Psychological disorders are important comorbidities for brain tumor patients [20, 21]. Emotional problems are simultaneously causing and indicating increased distress [8, 15, 16, 21]. In particular, depression and anxiety can impact quality of life [17, 22, 23]. (2) *Cognition* Cognition influences quality of life [16, 22, 24] and is associated with distress [11, 21] and unmet needs [16, 25]. Cognitive disorders can result from the tumor itself, therapy and associated symptoms, such as distress, anxiety, pain, depression, fatigue and sleep disturbances [23, 24, 26], as well as patient characteristics and supportive medication [20]. (3) *Body* Quality of life is influenced by physical complaints [3, 6, 25, 27], which are also associated with distress [8, 25, 27]. In glioma patients, seizures [20, 24, 26], weakness [5, 26], pain [5, 13, 20, 24], motor deficits [24], nausea/vomiting [24] and fatigue [13] are frequently reported symptoms. (4) *Role functioning* Impairments concerning work and leisure time can be related to distress and decreased quality of life [15, 21, 23, 25], although they are not consistently reported in the literature [9]. Particularly notable are financial burdens [25, 28] and limitations concerning the ability to work [23, 25]. (5) *Social support* Social problems can be associated with distress and decreased quality of life [16, 29] but not in a consistent manner [9], as the social wellbeing of glioma patients can even be higher than that of the standard population [25, 27]. Nonetheless, social problems are considered hardships that brain tumor patients face [23, 29]. (6) *Unmet needs* Quality of life assessment does not always indicate whether the individual patient needs help [30]. However, despite a high symptom load and high need for support, assessment remains demanding. Brain tumor patients may have difficulties detecting or expressing their needs and have limited access to assistant services [31].

### Interview conduction

All structured interviews were performed by the same interviewer in a separate room without presence of caregivers.

1. The patients answered whether the item personally affected them during the last week (yes/no), e.g., for psyche, one of the questions was “Were you recently sadder than you were before?—(yes/no)”. Subsequently, patients rated how important they perceived each item (with a six-point Likert scale ranging from 1 = not important to 6 = very important; the six-point scale was chosen to prevent a tendency toward the middle).
2. For each main area, patients were asked whether and if so, why they perceived items as missing or redundant.
3. Finally, patients rated the importance of the six main areas (on a six-point Likert scale) in general, e.g., for psyche the patients were asked: “At your own discretion, what score would you give ‘mood’ if 1 is the lowest score and indicates ‘unimportant’ and 6 is the highest score and indicates ‘important’?” Again, they were asked to indicate redundant or missing areas.

The interview in detail is provided in Supplementary File 1.

If patients experienced difficulties understanding a question during the interview, guidance was offered with the following three stepped scheme. *Step 1* The interviewer encouraged the patient to answer the respective question spontaneously and repeated the main topic of the question. *Step 2* The interviewer described an example of the topic or rephrased the sentence. *Step 3* The interviewer helped by repeating the main topic, rephrasing the sentence and giving an example. All occurring difficulties were documented.

### Health-care professionals’ survey

An online health-care professionals’ survey using SosciSurvey**®** was conducted. The same items applied in the patient interview were rated by the health-care professionals in terms of relevance (using a six-point Likert scale, see above). Different from the patients, the health-care professionals were asked to rank the main areas from 1 (most important) to 6 (least important). The following sociodemographic data were provided voluntarily by the colleagues: sex, age, profession, kind of hospital (e.g., university, maximum care hospital, private clinic), years of professional experience, and department/field.

### Statistics

#### Sample size

For a preliminary survey or scale development in a pilot study, a sample of “30 representative participants from the population of interest” is recommended [32]. We therefore aimed at least 30 analyzable patients’ interviews. Due to possible decliners or drop-outs, we included 50 patients and 36 health-care professionals were interviewed; such a sample size is also found in similar studies, e.g., Lai et al. with 50 patients and 10 health-care professionals [33].

#### Weighted scoring procedure

To select the three most important screening questions for our sample, a weighted scoring procedure was developed as decision guidance. According to recommendations of the EORTC quality of life group, patients’ personal relevance and importance ratings were taken into account. Patients should be given higher priority than health-care professionals [34]. Similar criteria can be found, e.g., for the assessment of the quality of care index in which the patients’ perceived reality and subjective importance are considered [35].

First, weighting was applied separately for the patients and health-care professionals. Then, the results of both groups were added up to generate a final score for each area.

**Fig. 2:**
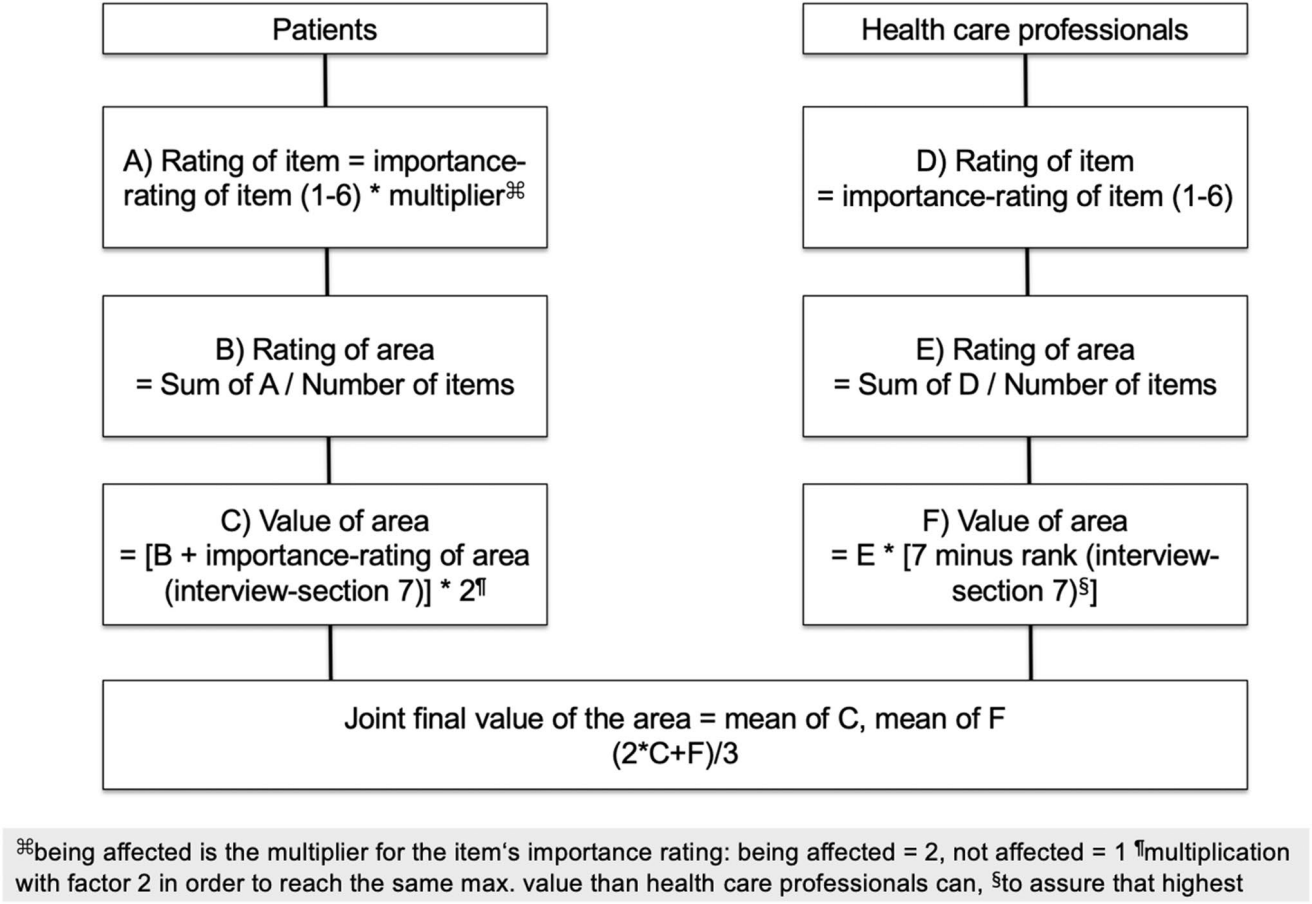
Weighted scoring procedure

The three areas with the highest final value were chosen for the screening questions that are a basis for further research to possibly be asked during the doctor–patient consultation. For each of the three screening questions, the most important item for patients (step A of the weighted scoring procedure) was additionally selected to be considered as a second question in the consultation. The steps of the weighted scoring procedure are shown in Fig. 2, and an example is given in Supplementary File 2.

## Results

### Patients and feasibility of the interview

From July 13th to October 12th, 2017, a total of 93 patients attending the neuro-oncological outpatient department at Mainz University Medical Center on interview days were considered for eligibility. Reasons for exclusion were lack of a glioma diagnosis (*n* = 15), insufficient understanding of the German language (*n* = 7) and the presence of only a relative without the patient (*n* = 2). A total of 69 patients met the inclusion criteria and were approached to participate. Reasons why patients declined were exhaustion (*n* = 8), time constraints (*n* = 4) and disinterest in participation (*n* = 6). Fifty-one patients were interviewed, and one patient was excluded during the interview because of severe language problems, resulting in the previously defined sample size of 50 patients. The median duration of the interview was 16.5 min (range 9–35 min). Approximately 26% of the patients needed no help during the entire interview, and 2 patients needed help with every item. Detailed information on the patient sample can be found in Table 1.

**Table 1:** Patient sample

Variable	*N* (50)	%100
Age		
Mean (SD, range)	56 (13, 30–79)	
Gender		
Male	35	70
Female	15	30
Family situation		
Married/ in a relationship	38	76
Divorced	4	8
Widowed	1	2
Single	7	14
Living situation		
Living alone	10	20
Living with others	40	80
Working situation		
Employed	12	24
Disability pension	22	44
Retirement pension	14	28
Homemaker	2	4
Graduation		
Lower secondary school	11	22
Intermediate secondary school	20	40
High school	19	38
Religion		
None	10	20
Evangelic	24	48
Catholic	13	26
Muslim	1	2
Free Church	1	2
Other	1	2
Side of tumor		
Right	21	42
Left	17	34
Midline	5	10
Multifocal	7	14
Last tumor resection prior to interview		
Gross total resection	37	74
Sub total resection	8	16
Biopsy	5	10
Chemotherapy during the last six weeks prior to the Interview		
No	30	60
Yes	20	40
Histopathology		
Glioblastoma, WHO Grade IV, IDH wildtype	23	46
Astrocytoma, WHO Grade III, IDH wildtype	3	6
Astrocytoma, WHO Grade III, IDH mutated	12	24
Astrocytoma, WHO Grade II, IDH mutated	2	4
Anaplastic Oligoastrocytoma^a^, NOS	3	6
Oligodendroglioma, WHO Grade III, IDH mutated	5	10
Ependymoma WHO Grade II	2	4
Localization of the tumor		
Frontal	15	30
Occipital	3	6
Parietal	9	18
Temporal	12	24
Others	11	22
Disease stage		
First diagnosis	31	62
Recurrence	19	38
Karnofsky performance score		
≥ 70	42	84
< 70	8	16
ECOG performance score		
0	13	26
1	22	44
2	15	30
Household net income in €/month		
Mean (SD, range)	2487.34 (1782.6; 400–8000)	
Time since diagnosis (months)		
Mean (SD, range)	62 (93; 0.7–369)	
Age at diagnosis (years)		
Mean (SD, range)	51 (16; 12–78)	

^a^Surgery before WHO 2016

### Health-care professionals’ survey

Members of the Neurooncology Working Group of the German Cancer Society (*n* = 350) were invited to participate in the anonymous online survey. Out of 38 participants (11%), a total of 36 were included in the analysis (one nonmedical practitioner and one person without information regarding their profession were excluded). Most health-care professionals were male neurosurgeons with more than 20 years of profession experience. Detailed information on the sample is provided as Supplementary Table 1.

### Selected screening and subordinate questions

The areas “cognition” (scoring value: 22.9), “body” (scoring value: 21.4) and “psyche” (scoring value: 18.9) were the three most relevant areas for both glioma patients and health-care professionals in our study. The final values of all areas after the weighted scoring procedure are shown in Table 2.

**Table 2:** Final values of the areas after the weighted scoring procedure

Area	Final value (joint)	Patients only (step C of the scoring procedure)	Health-care professionals only (step F of the scoring procedure)
Psyche	**18.9**	21.2	14.0
**Cognition**	**22.9**	23.9	21.0
**Body**	**21.4**	21.1	22.0
Role functioning	18.3	21.4	12.2
Social support	17.2	19.2	13.2
Unmet needs	15.5	18.0	10.4
Unmet needs calculated only with the top 5 items^a^	16.9	19.7	11.4

Bold values indicate most important areas according weighted scoring procedure

^a^The additional calculation was done because more precise selection of items for the interview considering literature could be done for the other areas compared to the area “unmet” needs”. To avoid that “unmet needs” had the lowest final value because of lesser item selection, it was calculated again using only the top 5 items of our patient and health-care professionals’ sample

For each area, the resulting screening question was formed by the authors as follows: The noun (e.g., “mood”) of the main area is mentioned directly in the questions with focus on changes for the worse (“has your mood worsened”). From the most important item of each area for our patient sample, a second question was derived:

1. *Screening question for psyche* “Has your mood worsened?” If the patient says “yes”, the most important item for the patient was “Are you unsure concerning the future?”
2. *Screening question for body* “Do physical changes put strain on you?” If the patient says “yes”, the most important item for the patient was “Do you have to rest more often because of exhaustion?”
3. *Screening question for cognition* “Has your memory capacity worsened?” If the patient says “yes”, the most important item for the patient was “Do you have difficulties concentrating, e.g., while reading a newspaper?”

### Comments of the health-care professionals and patients

After each main area, the patients and health-care professionals were asked whether they perceived questions as missing or redundant. After the importance rating of the main areas, they were also asked whether any main topics were dispensable or missing. Through these comments, we descriptively observed that some patients longed for more in-depth questions regarding the main area “psyche”, while other patients declared such questions redundant. Likewise, for the area “cognition” and “role functioning”, more precise questions were requested. Regarding “body”, a colleague remarked that one could see the obvious without asking. For “social support”, a question about neighborly relations was brought up. Considering “unmet needs”, an health-care professional raised the point of asking for unmet needs that possibly cannot be met. All comments are descriptively presented in Supplementary Table 2.

## Discussion

Adequately assessing the psychosocial distress and unmet needs of glioma patients can be demanding especially in the later disease trajectory when patients may not be able to be assessed more comprehensively by validated instruments any more. By conducting patient interviews, a health-care professional survey and subsequent decision guidance through a weighted scoring procedure, we selected three screening questions that may represent a basis for further research to improve the direct screening of glioma patients during patient–doctor consultation.

### Representativeness of the patient selection

In spite of the small sample size, we assume that the whole spectrum of glioma patients is represented in our sample. We included patients with gliomas representing the whole spectrum of histopathology and tumor localization according to literature [1].

### Selected screening questions and free comments of patients and health-care professionals

The high importance of “psyche” and the corresponding/subordinate item rated most important by patients (“uncertainty concerning the future”) is consistent with reports in the literature [23, 25, 28]. Some patients requested more in-depth questions regarding the main topic “psyche”, while some patients also suggested that these questions were redundant is in line with the results of the study by Sterckx et al. in which patients wished for more time to talk about their emotions and felt valued when staff took the time to do so but also emphasized the importance of staying optimistic [36].

The relevance subjective cognitive deficits have for glioma patients according to literature is also highlighted by the results of this study. It is, however, noticeable that the association between worse cognition and higher distress or decreased quality of life is not always consistent in the literature [37]. Patients requested more precise questions about memory for “cognition”, because memory decline represents an important source of frustration for glioma patients [38].

The importance of physical impairment (area “body”) and related problems for glioma patients is well known. The question about “fatigue” derived from the most important item rated by the patients reflects how frequently patients are affected by the symptom [21, 28, 29, 33, 39]. Fatigue is a complex symptom with multiple potential reasons [39]. Apart from the patients’ answers during the interview, it is also notable that the main reason eligible patients declined to participate in this study was because of exhaustion, which further highlights the importance fatigue also has for academic purposes. One colleague remarked that one could see the obvious without asking, which could reflect the skepticism of some health-care professionals toward screening [40]. On the other hand, glioma patients are observed at neuro-oncological outpatient centers closely and regularly. Although we recommend the “body” screening question according to our results, health-care professionals may recognize changes in clinical condition without asking patients directly.

Some patients asked for questions regarding alterations in role functioning. They are frequently reported in the literature; for instance, in a study by Piil et al. 75% of patients were active > 3 h/week before diagnosis, and only 9% had the same activity level at follow-up after one year [27].

Regarding “social support”, an additional question about neighborly relations could be considered as requested by patients.

Langbecker et al. noted that patients in their interview study had several complex needs for support [31], which is in line with the free comments in our study describing further requested topics. Furthermore, one health-care professional in our study discussed the point of asking about unmet needs that possibly cannot be met. On the one hand, this might be relevant when screening patients. Creating unmet needs by asking the patient should be done only when the needs can be addressed. The availability of psychosocial support options in different departments is highly variable despite certification procedures [31]. On the other hand, honest interest and asking per se is very important in the patient–doctor relationship. Feeling acknowledged can be a relief for patients.

Additionally, “information provision” was requested in our study and can also be found in other interviews. Patients and caregivers often long for more detailed or more adequate information [36, 38].

### Clinical implications

Especially for glioma patients with deficits, psychosocial assessment can be demanding since patients who are, e.g., not able to understand the structure of an instrument, to write or handle tablets due to any impairment may be missed by a screening. In this study, we generated three screening questions for glioma patients that are a basis for further research to possibly be asked during the doctor–patient consultation.

In general, using verbal screening questions may have some additional advantages:

It is known that clinicians can view screening as a burden [40]. Taking this skepticism into account, three questions during the patient–doctor consultation seem to be implementable into clinical routines.

Additionally, caregivers with a possibly alternative view of the patient’s distress and quality of life frequently fill in screening questionnaires for the patients and answers might be biased [3, 6], which could be avoided by directly asking the patients.

Not only in glioma patients but also in brain tumor patients in general, psychosocial and neurocognitive deficits may occur. Therefore, we hope that after further validation, the questions may even be applicable to other brain tumor patients after modification. Therefore, a multicenter study has been developed and is ongoing [41].

### Study limitations

The study design was explorative with a small sample size, pilot characteristics requiring further validation in a larger cohort of the resulting screening questions. Furthermore, we did not use a framework in order to select the six domains, which is a major limitation.

The interview, the survey and weighted scoring procedure used were specifically developed for this study. For the weighted scoring procedure, which was designed as decision guidance, it is notable that, using patient’s affection as multiplier for their importance rating, might introduce selection bias. However, the three selected items were also the three most important for the health-care professionals’ group. Furthermore, the questions are not validated so far and have to be translated in other languages.

A heterogeneous sample of glioma patients was included. Thus, on the one hand, influencing factors cannot be excluded. On the other hand, as outlined the heterogeneous group of patients visiting neuro-oncological outpatient clinics is represented in our patient sample.

Participating health-care professionals were mainly experienced male neurosurgeons, which could have influenced the survey.

## Conclusion

The three elaborated screening and subordinate questions could potentially be used along with validated instruments for psychosocial assessment adapted for glioma patients during doctor–patient consultations. Especially for patients in the late disease trajectory, the direct questioning may be an option instead of screening instruments or questionnaires. However, as they were developed in an explorative single-center study, further research with a multicenter investigation is required.

## Supplementary Information

Below is the link to the electronic supplementary material.

Supplementary Information 1 (DOCX 83 kb)

Supplementary Information 2 (DOCX 14 kb)

Supplementary Information 3 (DOCX 14 kb)

Supplementary Information 4 (DOCX 21 kb)

Supplementary Information 5 (DOCX 13 kb)

## Data Availability

The data that support the findings of this study are available from the corresponding author upon reasonable request.
