# Supplementary material for: Development of screening questions for doctor–patient consultation assessing the quality of life and psychosocial burden of glioma patients: an explorative study
Source: Qual Life Res. 2021 Jan 31;30(5):1513–22. doi: 10.1007/s11136-021-02756-x (PMC8068662; doi:10.1007/s11136-021-02756-x)
Supplement: Supplementary file 1 — Supplementary Information 1 (DOCX 83 kb) [file 11136_2021_2756_MOESM1_ESM.docx]

**Interview for patients**

1) Area „psyche“ / „mood“:

| **Items** | **Is the patient affected?** *„Referring to yourself, would you answer yes or no to the question about „x“? Please consider the last 7 days for that. („x“ = the item’s key point)“* | **Patients‘ importance rating**  *„At your own discretion, which point score would you give the question about „x“, if 1 is the lowest score meaning „unimportant“ and 6 is the highest score meaning „important“?* |
| --- | --- | --- |
| a) Were you recently sadder than you were before? | Yes No 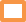 | 1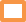 2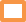 3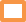 4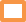 5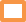 6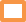 |
| b) Has your interest in things you normally enjoy decreased? | Yes 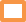 No 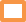 | 1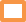 2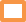 3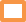 4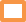 5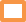 6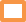 |
| c) Were you recently more tense than you were before? | Yes 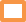 No 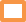 | 1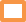 2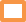 3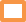 4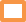 5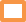 6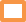 |
| d) Are you unsure concerning the future? | Yes 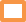 No 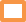 | 1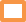 2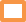 3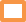 4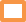 5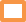 6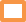 |
| e) Has your sleep behavior changed? | Yes 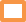 No 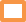 | 1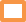 2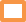 3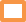 4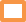 5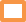 6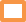 |

**Patients‘ comments:**
*„For the area „mood“ do you perceive questions as missing or redundant?“*

2) Area „cognition“ / „faculty of thought“

| **Items** | **Is the patient affected?** *„Referring to yourself, would you answer yes or no to the question about „x“? Please consider the last 7 days for that. („x“ = the item’s key point)“* | **Patients‘ importance rating**  *„At your own discretion, which point score would you give the question about „x“, if 1 is the lowest score meaning „unimportant“ and 6 is the highest score meaning „important“?* |
| --- | --- | --- |
| a) Do you remember things well? | Yes No 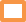 | 1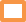 2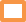 3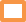 4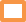 5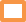 6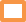 |
| b) Do you have difficulties to concentrate e.g. while reading a newspaper? | Yes No 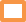 | 1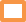 2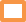 3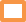 4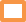 5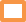 6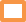 |

**Patients‘ comments:**
*„For the area „faculty of thought“ do you perceive questions as missing or redundant?“*


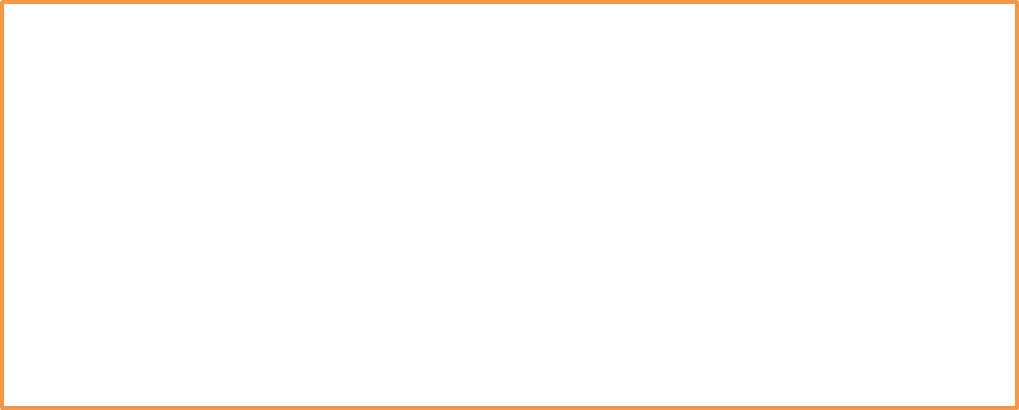


3) Area „body“ / „bodily impairments“

| **Items** | **Is the patient affected?** *„Referring to yourself, would you answer yes or no to the question about „x“? Please consider the last 7 days for that. („x“ = the item’s key point)“* | **Patients‘ importance rating**  *„At your own discretion, which point score would you give the question about „x“, if 1 is the lowest score meaning „unimportant“ and 6 is the highest score meaning „important“?* |
| --- | --- | --- |
| a) Is physical exertion problematic for you? | Yes No 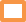 | 1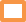 2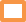 3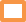 4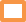 5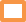 6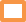 |
| b) Did you have pain (if yes: where?) | Yes 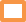 No 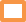  __________________________ __________________________ __________________________ __________________________ | 1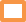 2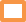 3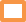 4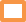 5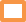 6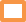 |
| c) Did you suffer from nausea or vomiting? | Yes No 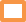 | 1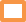 2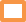 3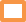 4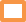 5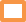 6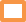 |
| d) Do you have to rest more often because of exhaustion? | Yes No 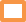 | 1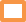 2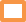 3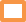 4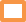 5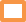 6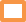 |
| e) Did you have epileptic seizures? | Yes No 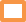 | 1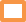 2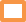 3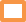 4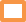 5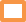 6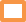 |
| f) Did you suffer from muscular weakness? | Yes No 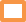 | 1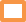 2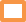 3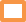 4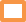 5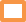 6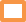 |
| g) Did you have coordination problems? | Yes No 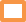 | 1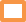 2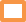 3 4 5 6 |
| h) Did you need assistance in everyday life e.g. while eating or having a wash? | Yes No | 1 2 3 4 5 6 |

**Patients‘ comments:**
*„For the area „bodily impairments“ do you perceive questions as missing or redundant?“*

4) Area “role functioning” / “work and leisure time”

| **Items** | **Is the patient affected?** *„Referring to yourself, would you answer yes or no to the question about „x“? Please consider the last 7 days for that. („x“ = the item’s key point)“* | **Patients‘ importance rating**  *„At your own discretion, which point score would you give the question about „x“, if 1 is the lowest score meaning „unimportant“ and 6 is the highest score meaning „important“?* |
| --- | --- | --- |
| a) Have financial problems developed? | Yes No | 1 2 3 4 5 6 |
| b) Did you have to reduce hobbies? | Yes No | 1 2 3 4 5 6 |
| c) Do you have problems with work? | Yes No | 1 2 3 4 5 6 |

**Patients‘ comments:**
*„For the area „work and leisure time“ do you perceive questions as missing or redundant?“*

5) Area “social support” / “relations to other people”:

| **Items** | **Is the patient affected?** *„Referring to yourself, would you answer yes or no to the question about „x“? Please consider the last 7 days for that. („x“ = the item’s key point)“* | **Patients‘ importance rating**  *„At your own discretion, which point score would you give the question about „x“, if 1 is the lowest score meaning „unimportant“ and 6 is the highest score meaning „important“?* |
| --- | --- | --- |
| a) Did you have problems with your family or partner? | Yes No | 1 2 3 4 5 6 |
| b) Did you have problems with other people outside of the family? | Yes No | 1 2 3 4 5 6 |

**Patients‘ comments:**
*„For the area „relations to other people“ do you perceive questions as missing or redundant?“*

6) Area “unmet needs” / “need of support”

| **Items** | **Is the patient affected?** *„Referring to yourself, would you answer yes or no to the question about „x“? Please consider the last 7 days for that. („x“ = the item’s key point)“* | **Patients‘ importance rating**  *„At your own discretion, which point score would you give the question about „x“, if 1 is the lowest score meaning „unimportant“ and 6 is the highest score meaning „important“?* |
| --- | --- | --- |
| a) Do you wish for more support from doctors? | Yes No | 1 2 3 4 5 6 |
| b) Do you wish for more support from physiotherapy? | Yes No | 1 2 3 4 5 6 |
| c) Do you wish for more support from a nursing service? | Yes No | 1 2 3 4 5 6 |
| d) Do you wish for more support from a social worker? | Yes No | 1 2 3 4 5 6 |
| e) Do you wish for more support from a psychologist? | Yes No | 1 2 3 4 5 6 |
| f) Do you wish for more support from a spiritual director or pastor? | Yes No | 1 2 3 4 5 6 |
| g) Do you wish for more support from nutrition counseling? | Yes No | 1 2 3 4 5 6 |
| h) Do you wish for more support from a self-helf group? | Yes No | 1 2 3 4 5 6 |
| i) Do you wish for more support from relatives? | Yes No | 1 2 3 4 5 6 |
| j) Do you wish for more support from friends? | Yes No | 1 2 3 4 5 6 |

**Patients‘ comments:**
*„For the area „need of support“ do you perceive questions as missing or redundant?“*

7) Importance rating of the areas
*„At last I wish to ask you to rate the areas in their importance. Once again you can give scoring points from 1 meaning “unimportant” to 6 meaning “important.”“*

| **Area** | **Patients‘ importance rating**  *„At your own discretion, which point score would you give the area „x“, if 1 is the lowest score meaning „unimportant“ and 6 is the highest score meaning „important“?* |
| --- | --- |
| a) Area “mood” | 1 2 3 4 5 6 |
| b) Area “faculty of thought” | *1 2 3 4 5 6* |
| c) Area “bodily impairments” | 1 2 3 4 5 6 |
| d) Area “work and leisure time” | 1 2 3 4 5 6 |
| e) Area “relations to other people” | 1 2 3 4 5 6 |
| f) Area “need of support” | 1 2 3 4 5 6 |

**Patients‘ comments:**
*„Did you perceive any of these areas as redundant or do you think that whole areas are missing?“*
