# Supplementary material for: Development of screening questions for doctor–patient consultation assessing the quality of life and psychosocial burden of glioma patients: an explorative study
Source: Qual Life Res. 2021 Jan 31;30(5):1513–22. doi: 10.1007/s11136-021-02756-x (PMC8068662; doi:10.1007/s11136-021-02756-x)
Supplement: Supplementary file 2 — Supplementary Information 2 (DOCX 14 kb) [file 11136_2021_2756_MOESM2_ESM.docx]

Supplementary file 2:

**Example of the weighted scoring procedure for “psyche”:**

Patients:

A) If a patient said “yes” to item “Were you recently sadder than you were before?”, his multiplier for this item is 2 (being affected = 2, not affected = 1). If he gave this item a score of 6 points, meaning it was an “important item”, the rating of the item (= importance-rating of item (1-6) * multiplier) would be 6 * 2 = 12.
B) The area psyche has 5 items. If the rating of each item (step A) was 12, the sum of A would be 5 * 12 = 60. The normalized rating of the area (= sum of A / number of items) would be 60 / 5 = 12.

C) We assume the patient gave the highest point score for “psyche” in the importance rating of the areas in the last interview section = 6 points. B + importance-rating of area (interview-section 7) would now be 12 + 6 = 18. To assure that the value of the area can be as high as the experts’ value, we multiplied by 2. The value of the area is therefore 18 * 2 = 36.

Health care professionals:

D) Since the rating of each item = importance-rating of item (1-6), if a health care professional gave the item “Were you recently sadder than you were before?” 6 points, the rating of the item = 6.

E) If the participant gave 6 points to every item of “psyche”, the normalized rating of the area (= sum of D / number of items) would be (5*6)/5 = 6.

F) In the last interview section, health care professionals were asked to rank all the areas from 1 = highest to 6 = lowest. To assure that the highest rank = most important area = the highest multiplier, we calculated with 7 minus the given rank (e.g., if the participant rated “psyche” rank 1 = most important, its value would be 6). The final value of the area (= E * (7-rank (interview-section 7)) = 6 * (7-1)) = 36.

Joint final value of “psyche”: (2*C+F)/3

Assuming all patients and all health care professionals answered similar to the example given above, the mean of C would be 36, and the mean of F would be 36 as well. To assure that patients are given higher priority, we multiply their value (C) by 2. The joint final value of “psyche” would be (2*36+36)/3 = 36.
