# Supplementary material for: Development of screening questions for doctor–patient consultation assessing the quality of life and psychosocial burden of glioma patients: an explorative study
Source: Qual Life Res. 2021 Jan 31;30(5):1513–22. doi: 10.1007/s11136-021-02756-x (PMC8068662; doi:10.1007/s11136-021-02756-x)
Supplement: Supplementary file 3 — Supplementary Information 3 (DOCX 14 kb) [file 11136_2021_2756_MOESM3_ESM.docx]

**Supplement Table 1: Health care professionals‘ Sample**

| **Variable** | **N (36)** | **%** |
| --- | --- | --- |
| **Gender**  Male  Female  Unknown | 30  5  1 | 83,3  13,9  2,8 |
| **Age**  36-45  46-55  >56  Unknown | 8  16  11  1 | 22,2  44,4  30,6  2,8 |
| **Years professional experience**  6-10  11-15  16-20  >20 | 1  6  9  20 | 2,8  16,7  25,0  55,6 |
| **Institution**  University Clinic  Hospital of maximum supply  Hospital of focus supply  Private Clinic  Others, please specify:  Medical service center at university clinic  private radiosurgical and radiotherapeutic institution | 20  11  2  1  1  1 | 55,6  30,6  5,6  2,8  2,8  2,8 |
| **Function**  Research  Clinical  Others, please specify:  both  Medical service center  Praxis, stereotactic radiation of brain tumors | 1  31  2  1  1 | 2,8  86,1  5,6  2,8  2,8 |
| **Field**  Neurology  Oncology  Radiooncology  Neurosurgery  Others, please specify:  Neurooncology  Palliative medicine | 9  1  4  20  1  1 | 25,0  2,8  11,1  55,6  2,8  2,8 |
