# Supplementary material for: Development of screening questions for doctor–patient consultation assessing the quality of life and psychosocial burden of glioma patients: an explorative study
Source: Qual Life Res. 2021 Jan 31;30(5):1513–22. doi: 10.1007/s11136-021-02756-x (PMC8068662; doi:10.1007/s11136-021-02756-x)
Supplement: Supplementary file 4 — Supplementary Information 4 (DOCX 21 kb) [file 11136_2021_2756_MOESM4_ESM.docx]

Supplementary Table 2: Patients' and health care professionals’ comments from the interviews and survey

| **PATIENTS‘ COMMENTS** |
| --- |
| **Area Psyche** |
| **Questions remarked as missing/ wishes to include:**   - Bodily condition - Relevance of the partner - Direct question how one’s feeling mentally - Influence of medication on sleep and psyche - Avolition - Prayer - Hair loss - Private problems - Do you need help mentally - Marred mood - Direct question about anxiety - Tiredness - Variations in feelings |
| **Questions remarked as dispensable/ redundant:**   - Loss of interests - Sadness (n=4) - Uncertainty concerning the future - Altered sleep - The questions are too superficial and need to be differentiated more |
| **Area Cognition** |
| **Questions remarked as missing/ wishes to include:**   - Logical intellectual capacity such as calculating - Has it always been like that - What exactly changed compared to the past - The topic psyche - Connection with the therapeutic situation - Are all thoughts clear - Do memory problems occur occasionally or all the time - Short time memory - Are coherent thoughts precise or confused - Is it hard to make decisions - How does one increase their ability to concentrate - Objective examination of the cognitive changes - Expressiveness (n=2) - Changed memories - Faculty of speech - Memory Retention |
| **Questions remarked as dispensable/ redundant:**   - The example reading a newspaper when questioning about concentration - The whole topic cognition - Memories need to be defined more clearly – negative or positive |
| **Area Body** |
| **Questions remarked as missing/ wishes to include:**   - Dealing with mental state - Is one able to manage housekeeping - How long ago was the operation and where exactly is the tumor located - Are there additional problems independent of the tumor - General condition - Vertigo attacks - Balance impairments (n=3) - It needs to be assessed what exactly the patients means with their answer as well as a question concerning motion sequence and support with car driving - The reason for nausea needs to be noted as well as nausea without vomiting considered and for epilepsy a question regarding genetic factors needs to be asked - Exhaustion |
| **Questions remarked as dispensable/ redundant:**   - Tiredness/Exhaustion - Help in everyday life such as eating and washing (n=2) |
| **Area Role Functioning** |
| **Questions remarked as missing/ wishes to include:**   - If one started new leisure activities now - More precise enquiries after the answer „yes“ and the importance rating - Reduced agility during leisure activities - How does the employment situation develop - Counselling concerning leisure time - Changes during the illness trajectory - The present situation in general - Child care and family life - Attitude to life - Does one regret not being able to work and does one have support concerning leisure activities - Does one exercise |
| **Questions remarked as dispensable/ redundant:**   - Hobbies - The whole topic (n=2) |
| **Area Social Support** |
| **Questions remarked as missing/ wishes to include:**   - The questions are asked too general and neighborly help is missing - What kind of support - Can others cope with the situation - How does one socialize in general - Early counseling concerning mental and physical well-being and counseling with the partner about side effects of the medication - Handling of family - Do neighbors behave differently |
| **Questions remarked as dispensable/ redundant:**   - The whole topic (n=3) |
| **Area Unmet Needs** |
| **Questions remarked as missing/ wishes to include:**   - Psychologist instead of Psychooncologist - Speech therapist - How outgoing and sociable one is - If one is religious - Support by the environment in general - Being lucky means having the people you need - What does one expect from the help - Need of support concerning employment situation - Need of financial counseling |
| **Questions remarked as dispensable/ redundant:**   - Pastor (n=5) - Friends - Social worker and nutrition counseling |
| **All areas in general** |
| **Topics remarked as missing/ wishes to include:**   - Including illness trajectory and asking if hedge is given - Practical tips for coping with the illness - Appetite - Helplessness during physician-patient-consultation and exchange of information - Economic situation - Information provision - Mood |
| **Topics remarked as dispensable/ redundant:**   - Mood - Unmet needs (N=2) - Body |

| **HEALTH CARE PROFESSIONALS‘ COMMENTS** |
| --- |
| **Comments on the area Psyche** |
| - Every patient is sadder, strained and uncertain - Timing of the questions is essential - Where there burdensome events in the family lately? - Contact with friends and family - Are you tired? - I ask questions more generally and not that specific - Increasing strains in the family - I ask about mood changes and thoughts and don’t anticipate negative content with my questions |
| **Comments on the area Cognition** |
| - Long- or short-term memory? - Patients do realize this less than family members do - Questions about organic brain changes are important, I don’t see a psychodynamic creation at the beginning |
| **Comments on the area Body** |
| - „Coordination problems“ should be framed more precisely - Maybe specify physical capacity and if eating or washing is meant - Some things are obvious and can be detected without asking |
| **Comments on the area Role Functioning** |
| - Question about occupation and dealing with set tasks, I prefer questions about leisure activities - Problems with partner, family and children |
| **Comments on the area Social Support** |
| - Those questions should rather be directed at environment and family |
| **Comments on the area Unmet Needs** |
| - What’s the point in asking about need of support that isn’t realizable? |
| **Comments on the areas in general** |
| No comments were made |
