# Supplementary material for: Development of screening questions for doctor–patient consultation assessing the quality of life and psychosocial burden of glioma patients: an explorative study
Source: Qual Life Res. 2021 Jan 31;30(5):1513–22. doi: 10.1007/s11136-021-02756-x (PMC8068662; doi:10.1007/s11136-021-02756-x)
Supplement: Supplementary file 5 — Supplementary Information 5 (DOCX 13 kb) [file 11136_2021_2756_MOESM5_ESM.docx]

| **Domain** | **Most important suordinate question of the domain for the patients**  (range: 1 – 12 points) |
| --- | --- |
| Psyche | Are you uncertain concerning the future? (Mean: 7.78) |
| Cognition | Do you have difficulties concentrating, e.g., while reading a newspaper (Mean: 7.18) |
| Body | Do you have to rest more often because of exhaustion? (Mean: 7.84) |

**Supplementary table 3:** Scores of the most important subordinate questions according tot he main domains
